# Untargeted Metabolomics Reveals Color-Dependent Nutritional Variation in Raisins: Insights into Composition and Antioxidant Capacity

**DOI:** 10.3390/antiox15030401

**Published:** 2026-03-23

**Authors:** Chuan Zhang, Shanwu Lyu, Vivek Yadav

**Affiliations:** 1 Institute of Fruits and Vegetables, Xinjiang Academy of Agricultural Sciences, Urumqi 830091, China; 2016204005@njau.edu.cn; 2 The State Key Laboratory of Genetic Improvement and Germplasm Innovation of Crop Resistance in Arid Desert Regions (Preparation), Urumqi 830091, China; 3 Key Laboratory of Genome Research and Genetic Improvement of Xinjiang Characteristic Fruits and Vegetables, Urumqi 830091, China; 4 Department of Biology, Queen’s University, Kingston, ON K7L 3N6, Canada; t2019113@njau.edu.cn

**Keywords:** non-targeted metabolome, differential metabolites, antioxidant capacity, syringetin

## Abstract

Raisins come from dried *Vitis vinifera* L. grapes. They are consumed worldwide, and their shape, color, texture, and taste largely determine consumer preference and market success. Consumers often select raisins based on visual appeal—namely color—without insight into how this relates to nutritional quality. Therefore, this study evaluated raisins of different colors based on non-targeted metabolomics to reveal the nutritional differences among differently colored raisins and to measure the differences in antioxidant capacity. Compared with green raisins (‘Sultanina’), 377–381 differential metabolites were identified in other colored varieties. Kyoto Encyclopedia of Genes and Genomes (KEGG) enrichment analysis revealed that these metabolites were enriched in pathways such as ‘biosynthesis of other secondary metabolites’ and ‘amino acid metabolism’. The comparison of the antioxidant capacity of raisins of different colors shows that the darker the color of the raisins, the stronger their antioxidant capacity. Correlation analysis between total antioxidant capacity and 14 differential metabolites showed a significant positive correlation. Notably, syringetin levels in black raisins (‘Blackcurrant’ and ‘Sweet Sapphire’) were substantially higher—148.31 and 515.94 times greater, respectively—than in green raisins (‘Sultanina’). This elevated syringetin content may significantly contribute to the enhanced antioxidant capacity of black raisins. Furthermore, based on the positive ion mode, the relative contents of 24 and 12 differential metabolites were relatively high in green and red raisins, respectively. The negative ion model identified that 19 and 4 differential metabolites had relatively high contents in green and red raisins. These metabolites may be linked to the unique health benefits of red and green raisins. This study provides valuable insights for consumers selecting raisins based on health needs and for companies developing raisin-based health products.

## 1. Introduction

Grapes are a well-known fruit and have been cultivated worldwide for a long time. Grape berries have a wide variety of flavors and are rich in various vitamins and polyphenols, such as proanthocyanidins [1], resveratrol [2], and anthocyanins [3]. Grapes are very popular in the market because of their taste and health benefits. The area of the world’s vineyards has been gradually increasing. As of 2023, it has reached 7,141,570 ha. Meanwhile, the output of fresh grapes has continued to increase. As of 2023, it reached 74,684,654 tons. Among them, the production of table grapes reached 31,934,318 tons. The production of dried grapes reached 1,154,835 tons. This is largely due to consumers’ demand for seedless grapes for fresh and dried berry consumption [4]. In 2023 alone, the global consumption of table grapes and dried grapes was 31,713,166 tons and 1,224,190 tons, respectively [4]. Although the market for fresh grapes is vast, due to various factors, including climatic conditions, fluctuations in market supply and demand, the diversity of product choices, and the deep processing of grapes to enhance their commercial value, a considerable portion of grapes are processed into raisins.

Raisins belong to the traditional category of dried fruits because they usually do not contain added sugar. Approximately 95% of the raisins are dried ‘Thomson seedless’ (Sultanina) grapes [5]. The history of raisins can be traced back to prehistoric times when grapes were dried for storage and transportation. There is evidence that raisins have been used for consumption for a long time. In the Mediterranean region, it is a frequently consumed food and one of the dried fruits characteristics of the Mediterranean diet. Furthermore, it is widely used as traditional and natural biomedicine in some climate-adapted countries [6].

Interestingly, the color and variety of raisins depend on the type of dried grapes. These are cheap all year round and popular worldwide and are usually used for cooking salty and sweet dishes [7]. Raisins are rich in sugar (glucose, fructose, and sucrose), vitamins (ascorbic acid, pyridoxine, riboflavin, and thiamine), dietary fiber, minerals (potassium, sodium, magnesium, and iron), and the trace element boron [8]. The sugar content of raisins and grapes is approximately the same, the contents of fructose and glucose are almost equal, and the content of sucrose is the least [9]. The sweetness of raisins is attributed to the presence of glucose and fructose. Raisins are rich in dietary fiber (3.3–4.5 g per 100 g), which contributes to their prebiotic effects [10] because they are selectively used by host microorganisms and confer health benefits [11]. In addition, raisins are rich in various polyphenols that are particularly beneficial to human health. Among them, the most abundant were flavonols (quercetin and kaempferol derivatives) and phenolic acids (mainly caftaric and coutaric acid). Most of the compounds present in raisins come from fresh grapes, but other compounds increase during processing, such as caffeoyl tartaric acid and some quercetin and kaempferol derivatives [9]. Studies have pointed out that raisins contain more abundant certain acids, such as protocatechuic acid and oxidized cinnamic acid, than their hydrates [12].

In order to better measure the aroma components in raisins, researchers have employed a variety of advanced measurement techniques [13,14,15]. Some studies have used Headspace Solid-Phase Microextraction (HS-SPME) and Gas Chromatography–Mass Spectrometry (GC-MS) to investigate the post-storage changes of volatile compounds in air-dried and sun-dried raisins from different packaging materials and determined that fatty acid oxidation (UFAO) and Maillard reaction (MR) compounds are the main sources of the aroma of raisins [15]. HS-SPME with GC-MS was used to detect volatile compounds of raisin-related unsaturated fatty acids. All fatty acid (FA) reactions are the reasons for generating more concentrated fatty acid oxidation (UFAO) derivatives as volatile substances [13]. At present, among the various studies on the sensory and nutritional qualities of raisins, the research on aroma is the most extensive. In addition, the amino acids in the grape dry varieties were analyzed using an Agilent high-performance liquid chromatograph (HPLC) (Agilent Technologies, Santa Clara, CA, USA); the research found that the content of most amino acids in seeded raisin varieties was higher than that in raisins produced by seedless varieties [16]. Relatively speaking, the determination of raisins’ comprehensive nutritional components is relatively scarce, which has greatly hindered people’s understanding of the nutritional components of raisins [17]. The core significance of metabolomics analysis lies in precisely identifying the types and content changes of active substances in raisins, providing a scientific basis for the development of functional foods and healthy consumption. Some researchers have attempted to use a metabolomics analysis system to investigate the regulatory effects of different Light Emitting Diode (LED) light treatments on the composition of metabolites during the drying process of seedless white grapes. Purple LED light mainly promotes the accumulation of functional components by activating secondary metabolite biosynthesis and amino acid metabolic pathways, thereby achieving a comprehensive improvement in nutritional quality. This study provides a theoretical basis and technical path for enhancing the nutritional value and processing quality of grape dried products [18].

Interestingly, the color and variety of raisins depend on the type of dried grapes. Traditionally, the dried grape varieties in Xinjiang, China, which produces about 95% of the country’s raisins, have mainly been green. In recent years, however, the introduction and wider cultivation of new grape varieties have expanded the range from the traditional green to include dark green, yellowish green, black, red, and other colors. Currently, the main raisin varieties available on the market include green raisins (‘Sultanina’ and ‘Green Xiangfei’), red raisins (red ‘Xiangfei’), and black raisins (‘Black Beauty’, ‘Blackcurrant’, and ‘Sweet Sapphire’). Studies have showed that the neuroprotective effect of raisins is attributed to the moderate consumption of grape polyphenols [19]. The main phenolic compounds in red grapes are flavan-3-ols (catechin, epicatechin, proanthocyanidins B1, B2, and C1, and gallocatechin). To a lesser extent, both red and white grapes contain hydroxybenzoic acids, hydroxycinnamic acids, stilbenes (such as resveratrol), and flavonols (especially rutin), as well as derivatives of quercetin glycosides [20]. The diversity of this compound makes grapes, especially red grapes, an excellent candidate for testing the role of dietary polyphenols in health.

Xinjiang raisins are available in a wide variety of types, each with distinct characteristics. Consumers can select raisins based on their preferences and nutritional needs. However, many consumers lack clear guidance when choosing raisins. Therefore, this study employs non-targeted metabolomics to investigate the differences in nutritional components and antioxidant capacities among raisins of various colors. Our research findings indicate that black raisins have high levels of (−)-epigallocatechin and myricetin. These substances may contribute to their excellent total antioxidant capacity and potential as functional foods to reduce the risk of ROS-related diseases. Green or brown raisins contain relatively high levels of substances that may have special functions and require further in-depth research. The findings aim to provide evidence-based guidance for consumers to select raisins that best meet their dietary and health requirements.

## 2. Materials and Methods

### 2.1. Raisin Sample Collection

The experimental varieties for this study consisted of four raisin samples procured from our industrial partner in Xinjiang, China, with products marketed through the Hualing Dried Fruit Comprehensive Wholesale Market. These samples included red raisins (‘Hongxiangfei’), black raisins (‘Blackcurrant’ and ‘Sweet Sapphire’), and green raisins (‘Sultanina’). All varieties were cultivated at the same research site under identical environmental and agricultural conditions (irrigation, fertilization, and pest management) and processed using standardized drying protocols. Specifically, all varieties were planted in the grape planting base of the Xinjiang Uygur Autonomous Region Grape and Fruit Research Institute (42°54′41″ E, 90°17′17″ N). All varieties were cultivated using a horizontal trellis with multiple vines, and the column height was 2.0 m. The spacing between plants was 4.0 m × 0.5 m. All varieties used the same production process for raisins. Good quality berries were selected and rinsed with clean water to remove dust and impurities, followed by mechanical removal of fruit stems. The grapes were then soaked in a solution of 0.2% sodium hydroxide for 5 min to break down the wax layer of the skin and accelerate water evaporation, and subsequently thoroughly rinsed. The microwave raisin dryer (VYS-30HO; tunnel type) produced by Guangzhou Weiyasi Microwave Equipment Co., Ltd. (Guangzhou, China) was used for rapid drying of cleaned raisins, with simultaneous microwave sterilization and insecticidal treatment. After continuous drying for 72 h, the moisture content of raisins dropped to around 15–20%, which was the end point of drying. Vacuum packaging was used to extend the shelf life of raisins. This ensured consistency in baseline metabolite profiles and eliminated potential confounding variables associated with multi-source sourcing. To preserve their integrity for subsequent analyses, all samples were stored at −80 °C prior to metabolomics profiling and evaluation of antioxidant activity.

### 2.2. Determination of Raisins’ Phenotypic Traits

Ten raisins were randomly selected, and the weight of each dried grape was measured using an electronic balance. The average weight per raisin (in grams) was then calculated. Additionally, ten raisins were randomly selected, and their longitudinal and transverse diameters were measured using a vernier caliper. The average longitudinal and transverse diameters (in millimeters) for each raisin were subsequently determined.

### 2.3. Determination of Total Antioxidant Capacity

Raisin samples of different colors were ground into a fine powder. A 0.1 g portion of each sample was weighed, and 0.5 mL of 80% methanol was added. The mixture was vortexed to ensure thorough mixing and then incubated in a water bath at 60 °C for 30 min. Subsequently, the mixture was centrifuged at 10,000× *g* for 10 min at room temperature, and the supernatant was collected for analysis.

For the DPPH assay, we followed the method described by Adiletta et al. [21], with some modifications. Namely, 10 µL of the supernatant was mixed with 190 µL of a 0.1 mmol/L 1,1-diphenyl-2-picrylhydrazyl (DPPH) solution in anhydrous ethanol. The mixture was thoroughly mixed and incubated at room temperature in the dark for 30 min. After incubation, it was centrifuged at 8000× *g* for 10 min at room temperature. A 200 µL aliquot of the resulting supernatant was used to measure absorbance at 515 nm. For the control, the DPPH solution was replaced with anhydrous ethanol. For the blank, the supernatant was replaced with an 80% methanol solution. For the positive control, the supernatant was replaced with vitamin C solutions of varying concentrations.

The calculation formula for the free radical scavenging rate of the VC-positive control:

The free radical clearance rate of DPPH (DVC%) = [A_blank_ – A_positive control_)/A_blank_] × 100%.

The calculation formula for the free radical scavenging rate of the sample:

The free radical scavenging rate of DPPH (D%) = [A_blank_ – (A_determination_ – A_control_)]/A_blank_] × 100%

The calculation formula for the free radical scavenging capacity of the sample:

Total antioxidant capacity (U/g) = D% × V_extraction_ × V_total reaction volume_/V_sample/_M × F

In these formulas: D%—the free radical clearance rate of DPPH in the sample; V_total reaction volume_—total reaction volume, 0.2 mL; V_sample_—the volume of the sample in the reaction, 0.01 mL; V_extraction_—the added volume of the extraction liquid, 0.5 mL; M—sample mass, g; and F—dilution factor.

Unit definition: When the clearance rate in the above reaction system is greater than 5%, the free radical scavenging capacity in the reaction system is defined as one unit.

### 2.4. Determination of Polyphenol Oxidase (PPO) Activity

For the PPO activity assay, we followed the method described by González et al. [22] and Tang and Newton [23], with some modifications. Raisin samples of different colors were ground into a fine powder. Approximately 0.1 g of each sample was weighed and mixed with 0.5 mL of phosphoric acid buffer solution. The mixture was thoroughly vortexed to ensure homogeneity. Subsequently, 0.1 mL of catechol solution was added and mixed well, and the samples were subjected to an oxidation reaction in a constant-temperature shaker at 37 °C for 15 min. The reaction was terminated by rapidly transferring the samples to an ice-water bath at 0 °C for 3 min. The samples were then centrifuged at 10,000× *g* for 10 min at 4 °C. Then, 200 µL of the supernatant was taken and the absorbance was measured at 410 nm, which is recorded as A_determination_.

PPO activity (U/g) = A_determination_/0.01/M/T × F

In this formula: T—reaction time, 15 min; M—sample mass, g; and F—dilution factor.

Unit definition: A change of 0.01 in absorbance value per g of sample per minute is defined as one unit of enzyme activity.

### 2.5. Determination of Hydroxyl Radical Scavenging Capacity

For the hydroxyl radical scavenging capacity assay, we followed the method described by Takeshi and Rciji [24], with some modifications. Raisin samples of different colors were ground into a fine powder. A 0.1 g portion of each sample was weighed and mixed with 0.5 mL of phosphate-buffered saline (PBS) solution. After thorough homogenization, the mixture was centrifuged at 10,000 rpm for 10 min at room temperature, and the supernatant was collected for analysis. For the Fenton reaction assay, 50 µL of 6 mmol/L ferrous sulfate solution, 50 µL of 6 mmol/L hydrogen peroxide solution, 50 µL of 6 mmol/L salicylic acid-ethanol solution, and 50 µL of the test supernatant were sequentially added to a 96-well plate. The mixture was thoroughly mixed, and the absorbance was measured at 510 nm, recorded as A_determination_. For the control, distilled water was used in place of the hydrogen peroxide solution. For the blank, distilled water was used in place of the sample supernatant. All measurements were performed simultaneously with the test solution.

The absorbance value is recorded as A_control_ and A_blank_.

Hydroxyl radical scavenging rate (D%) = [A_blank_ – (A_determination_ – A_control_)]/A_blank_ × 100%.

### 2.6. Determination of the Superoxide Anions Scavenging Capacity

For the superoxide anions scavenging capacity assay, we followed the method described by Lu et al. [25], with some modifications. Raisin samples of different colors were ground into a fine powder. Approximately 0.1 g of each sample was weighed and mixed with 0.5 mL of distilled water. After thorough mixing and homogenization, the samples underwent ultrasonic extraction for 30 min. The mixture was then centrifuged at 12,000× *g* for 10 min at 25 °C, and the supernatant was collected for analysis.

For the superoxide anion scavenging assay, 100 µL of buffer solution, 80 µL of the test supernatant, and 10 µL of catechol solution were combined and mixed thoroughly. The mixture was incubated at 25 °C for 4 min, and the absorbance was measured at 325 nm, recorded as A_determination_. For the control, distilled water was used in place of the catechol solution, and the absorbance was recorded as A_control_. For the blank, distilled water was used in place of the sample supernatant, and the absorbance was recorded as A_blank_. All measurements were performed simultaneously with the test solution.

Superoxide anions scavenging capacity (D%) = [A_blank_ – (A_determination_ – A_control_)]/A_blank_ × 100%

### 2.7. Extraction of Samples for Metabolite Analysis

Raisin samples of different colors were accurately weighed (8.0 g each) and placed into separate 50 mL polytetrafluoroethylene centrifuge tubes. Metabolites were extracted using 20 mL of each of three solvents—ethyl acetate, acetonitrile, and methanol—separately for each sample [26]. Each sample was homogenized with a homogenizer for 1 min and then centrifuged at 4000 rpm for 5 min. The supernatant was collected and transferred to a 100 mL brown conical flask. The supernatant was evaporated in a water bath at 40 °C using a rotary evaporator until nearly dry and then completely dried under a nitrogen stream. The residue was dissolved in 1.0 mL of one of the following solvents: 100% methanol, 100% acetonitrile, 50% methanol, or 50% acetonitrile [27]. After thorough vortex mixing, the solution was filtered through a 0.22 μm organic phase filter membrane into a sample vial for analysis.

### 2.8. Ultra-Performance Liquid Chromatography–Quadrupole Time-of-Flight Mass Spectrometry

Raisin samples of different colors were analyzed using an Agilent ZORBAX column (2.1 mm × 100 mm, 1.8 µm; Agilent Technologies, Santa Clara, CA, USA) for chromatographic separation. In positive ion mode, the mobile phase consisted of an aqueous solution with 0.1% formic acid (mobile phase A) and an acetonitrile solution with 0.1% formic acid (mobile phase B), while in negative ion mode, mobile phase A was an aqueous solution with 0.1% acetic acid, and mobile phase B was an acetonitrile solution. The injection volume was 5 µL, with a flow rate of 0.3 mL/min and a column temperature maintained at 40 °C. The elution gradient was programmed as follows: 0–3 min at 10% B, 3–12 min increasing from 10% to 40% B, 12–14 min at 40% B, 14–17 min increasing from 40% to 95% B, 17–17.5 min at 95% B, 17.5–19 min decreasing from 95% to 10% B, and 19–20 min at 10% B. Data were acquired in both positive and negative ion modes using a QTOF mass spectrometer equipped with an ESI ion source (QTOF X500; AB SCIEX, Framingham, MA, USA), with a 50% methanol solution as the blank control [28]. The mass range was set to *m*/*z* 40–1200 in full scan mode. Instrument parameters for positive and negative ESI modes included a spray voltage of +5500 V and −4500 V, respectively; a declustering potential of +80 V and −80 V; a source temperature of 550 °C; and collision energies of +35 eV and −35 eV with a ±15 eV spread. Gas settings were configured as follows: curtain gas pressure at 30 psi, nebulizer gas (GAS1) at 50 psi, and auxiliary heating gas (GAS2) at 55 psi. An automatic calibration system was utilized, calibrating every five samples to ensure analytical accuracy and reproducibility [29].

### 2.9. Data Processing and Identification of Metabolites

Raw mass spectrometry data were converted to mzXML format using the MSConvert tool (version 3.0) from the ProteoWizard software suite. Peak extraction and quality control (QC) were performed using XCMS software (version 4.8.0), with low-quality peaks removed based on missing value thresholds exceeding 50% in QC samples or 80% in actual samples. Missing values were imputed using K-nearest neighbors (KNN) interpolation. The filtered dataset was obtained by excluding ions with a coefficient of variation (CV) greater than 30% across all QC samples. Extracted substances were annotated using the CAMERA package (version 1.66). The filtered data were then analyzed in SIMCA-P software (version 12.0.1) through unsupervised principal component analysis (PCA). Univariate statistical analysis was conducted, including fold change and *t*-test, with Q values calculated using the Benjamini–Hochberg (BH) method for multiple testing correction. Multivariate statistical analysis was performed using partial least squares discriminant analysis (PLS-DA) to determine the variable importance in projection (VIP) values for predictor variables.

## 3. Results

### 3.1. Comparison of the Phenotypes of Raisins with Different Colors

**Figure 1:**
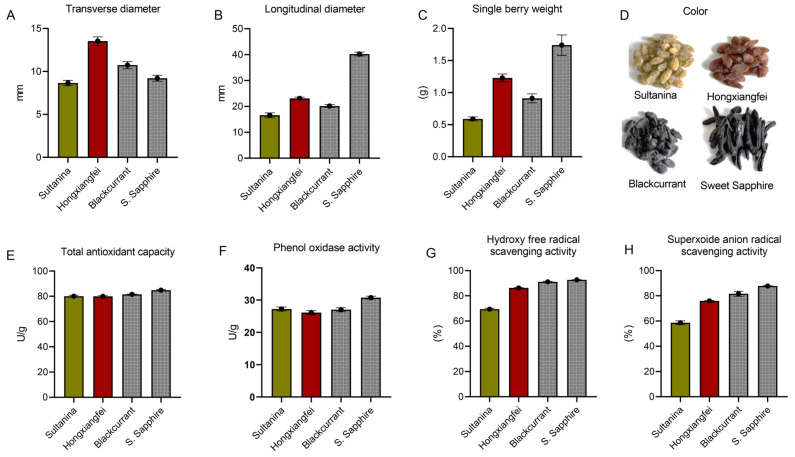
Comparison of phenotypic and antioxidant capacity of raisins with different colors. (**A**) Transverse diameter (mm), (**B**) longitudinal diameter (mm), (**C**) single-berry weight (g), (**D**) true color of berries, (**E**) total antioxidant capacity (U/g), (**F**) phenol oxidase activity (U/g), (**G**) hydroxy free radical scavenging activity (%), and (**H**) superoxide anion radical scavenging activity (%).

The phenotypic comparison of raisins of different colors is shown in Figure 1 . Based on measured data, the red raisin cultivar ‘Hongxiangfei’ exhibits the largest transverse diameter (Figure 1A), and the black raisin cultivar ‘Blackcurrant’ ranks second, while the green raisin cultivar ‘Sultanina’ has the smallest transverse diameter. The black raisin ‘Sweet Sapphire’ has the largest longitudinal diameter, followed by the red raisin ‘Hongxiangfei’ (Figure 1B). The green raisin ‘Sultanina’ has the smallest longitudinal diameter. The black raisin ‘Sweet Sapphire’ has the largest single-grain weight, while the red raisin ‘Hongxiangfei’ has the second largest (Figure 1C). The green raisin ‘Sultanina’ has the smallest single weight. The original color of the raisin varieties is shown in Figure 1D.

### 3.2. Comparison of Antioxidant Capacity of Raisins with Different Colors

The comparison of antioxidant capacity among raisins of different colors is presented in Figure 1. Overall, the antioxidant capacity of black raisins (Blackcurrant and Sweet Sapphire) is significantly higher than that of red raisins (Hongxiangfei) and green raisins (Sultanina) (Figure 1E). Furthermore, the antioxidant capacity of red raisins (Hongxiangfei) is significantly greater than that of green raisins (Sultanina). Among all samples, the black raisin Sweet Sapphire exhibited the highest values for total antioxidant capacity (Figure 1E), polyphenol oxidase activity (Figure 1F), hydroxyl radical scavenging capacity (Figure 1G), and superoxide scavenging capacity (Figure 1H), reaching 84.87 U/g, 30.76 U/g, 92.67%, and 87.75%, respectively. In contrast, the green raisin ‘Sultanina’ recorded the lowest values for these parameters, measuring 80.03 U/g, 27.23 U/g, 69.38%, and 58.61%, respectively.

### 3.3. Distribution of Differential Metabolites of Raisins of Different Colors in Different Pathways

**Figure 2:**
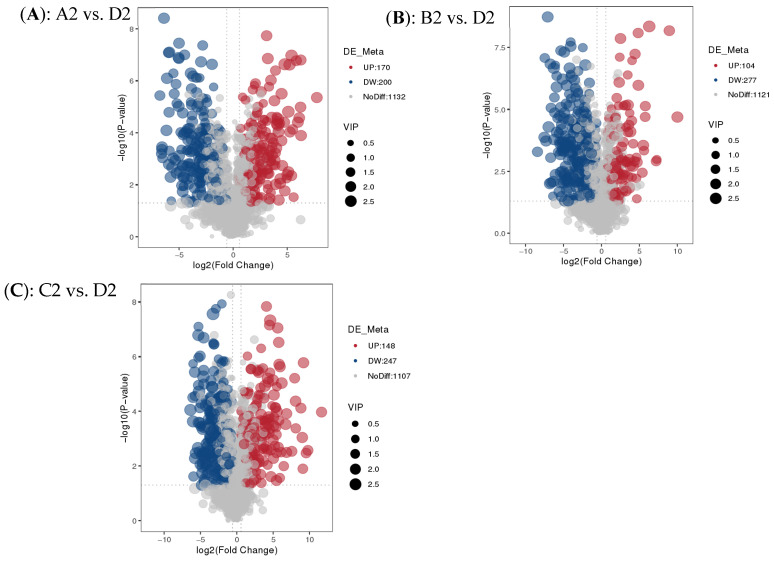
Volcano plots showing differentially abundant metabolites identified across three variety comparisons: (**A**) A2 vs. D2, (**B**) B2 vs. D2, and (**C**) C2 vs. D2. Red and blue dots represent significantly upregulated (UP) and downregulated (DW) metabolites, respectively, while gray dots indicate non-differentially abundant metabolites (NoDiff). Dot size corresponds to Variable Importance in Projection (VIP) scores, with larger circles indicating higher VIP values. (A2: ‘Hongxiangfei’; B2: ‘Blackcurrant’; C2: ‘Sweet Sapphire’; D2: ‘Sultanina’).

The analysis of the differences in metabolites of raisins with different colors is shown in Figure 2. It can be observed from Figure 2A that 370 kinds of metabolites with significant differences between ‘Hongxiangfei’ and ‘Sultanina’ raisins were identified (Table S1). There were 170 kinds of significantly up-regulated metabolites and 200 kinds of significantly down-regulated metabolites. It can be seen from Figure 2B that the number of metabolites with significant differences between ‘Blackcurrant’ and ‘Sultanina’ raisins identified is 381 kinds. There were 104 kinds of metabolites significantly up-regulated and 277 kinds of metabolites significantly down-regulated. It can be seen from Figure 2C that the number of metabolites with significant differences between ‘Sweet Sapphire’ and ‘Sultanina’ raisins identified is 395 kinds. There were 148 kinds of metabolites significantly up-regulated and 247 kinds of metabolites significantly down-regulated. The correlation analysis of the differential metabolites of raisins with different colors identified is shown in Figures S1–S3. It can be concluded that the high correlation coefficients of the top 20 metabolites indicate the existence of specific coordinated regulatory metabolic clusters.

**Figure 3:**
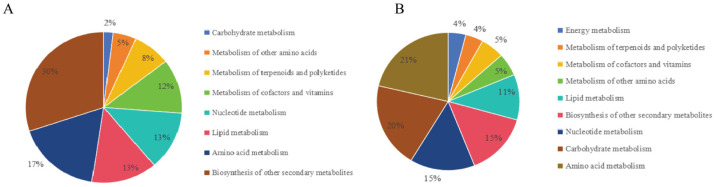
The quantitative distribution of differential metabolites of raisins with different colors in different pathways. Note: (**A**) positive ion mode; (**B**) negative ion mode.

The distribution of differential metabolites among the raisins ‘Hongxiangfei’, ‘Blackcurrant’, ‘Sweet Sapphire’, and ‘Sultanina’ across different pathways is shown in Figure 3. Under the positive ion mode (Figure 3A), the highest proportion was enriched in ‘biosynthesis of other secondary metabolites’ (30%), followed by ‘amino acid metabolism’ (17%), with ‘carbohydrate metabolism’ showing the lowest proportion (2%). Under the negative ion mode (Figure 3B), amino acid metabolism, accounted for the largest share (21%), followed by ‘carbohydrate metabolism (20%), whereas ‘energy metabolism’ and ‘terpenoid metabolism’, and polyketides’ each represented only 4% of the total.

### 3.4. Differential Metabolite Profiles in Positive Ion Mode

**Figure 4:**
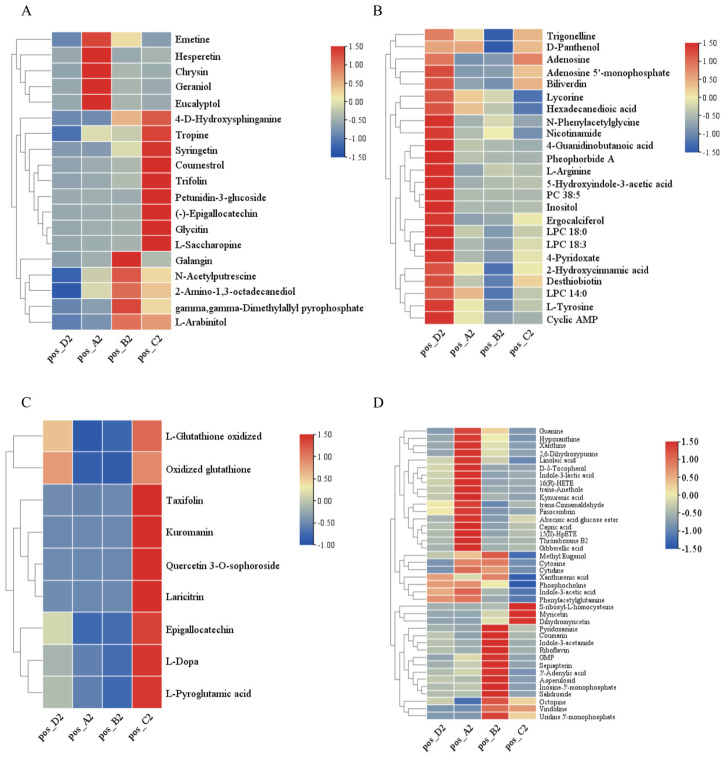
(**A**–**D**) The relative content differences of differential metabolites in raisins with different colors identified based on the positive ion mode. Notes: A2: ‘Hongxiangfei’; B2: ‘Blackcurrant’; C2: ‘Sweet Sapphire’; D2: ‘Sultanina’.

Based on the positive ion mode, a total of 92 differential metabolites were identified among raisins of different colors (Figure 4). In Figure 4A, 19 metabolites were significantly more abundant in red (Hongxiangfei) and black (Blackcurrant, Sweet Sapphire) raisins than in green (Sultanina). Syringetin content in Blackcurrant and Sweet Sapphire was 148.31 and 515.94 times higher, respectively, than in Sultanina. In Figure 4B, 24 metabolites (including 2-hydroxycinnamic acid, L-tyrosine, L-arginine, lycorine, N-phenylacetylglycine, 4-guanidinobutanoic acid, 5-hydroxyindole-3-acetic acid, various LPCs, ergocalciferol, adenosine derivatives, trigonelline, nicotinamide, desthiobiotin, 4-pyridoxate, D-panthenol, biliverdin, pheophorbide A, and inositol) were significantly lower in red and black raisins than in green raisins. Inositol content in Hongxiangfei, Blackcurrant, and ‘Sweet Sapphire’ was only 0.03-, 0.01-, and 0.05-fold that of Sultanina, respectively. In Figure 4C, nine metabolites were significantly higher in Sweet Sapphire than in the other cultivars, with quercetin 3-O-sophoroside, laricitrin, and kuromanin being 31.34-, 32.75-, and 544.52-fold higher than in Sultanina, respectively. In Figure 4D, 40 additional metabolites showed varied distribution patterns. Seven compounds (e.g., myricetin, dihydromyricetin, vindoline, and octopine) were more abundant in ‘Blackcurrant’ and ‘Sweet Sapphire’ than in ‘Hongxiangfei’ and ‘Sultanina’; myricetin and dihydromyricetin were up to 115.64- and 61.77-fold higher, respectively, than in ‘Sultanina’. Eleven nucleotide metabolites were higher in ‘Hongxiangfei’ and ‘Blackcurrant’ than in ‘Sweet Sapphire’ and ‘Sultanina’. Twelve metabolites were higher in ‘Hongxiangfei’ than in all other cultivars. Six metabolites (e.g., coumarin, salidroside, and riboflavin) were highest in ‘Blackcurrant’, and four metabolites (e.g., capric acid and gibberellic acid) were higher in ‘Hongxiangfei’ and ‘Sweet Sapphire’ than in the remaining cultivars.

### 3.5. Differential Metabolite Profiles in Negative Ion Mode

**Figure 5:**
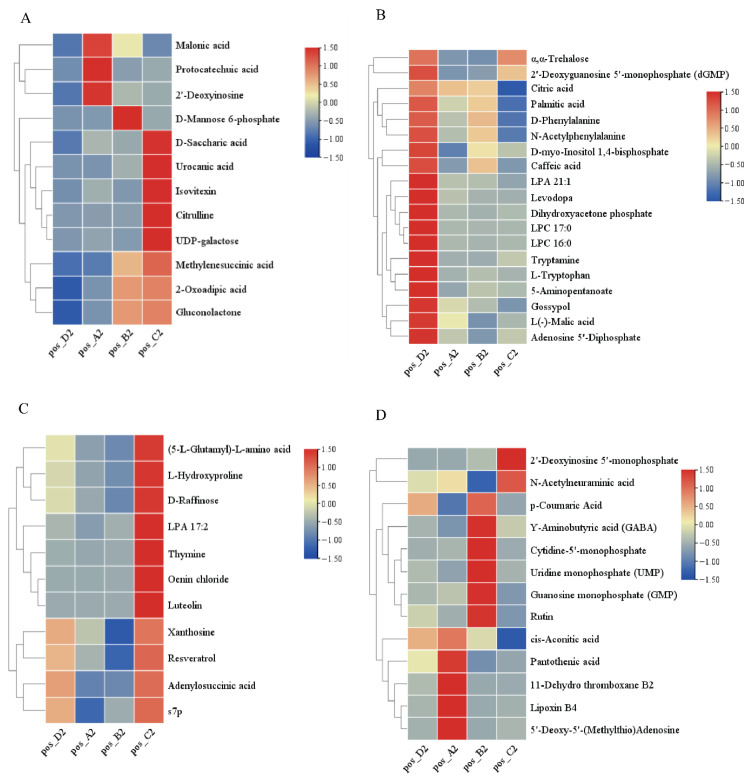
(**A**–**D**) The relative content differences of differential metabolites in raisins with different colors identified based on the positive ion mode. Notes: A2: ‘Hongxiangfei’; B2: ‘Blackcurrant’; C2: ‘Sweet Sapphire’; D2: ‘Sultanina’.

Based on the negative ion mode, 55 differential metabolites were identified among raisins of different colors (Figure 5). In Figure 5A, 12 metabolites (2-oxoadipic acid, citrulline, protocatechuic acid, urocanic acid, methylenesuccinic acid, gluconolactone, D-mannose 6-phosphate, UDP-galactose, malonic acid, 2′-deoxyinosine, isovitexin, and D-saccharic acid) were significantly more abundant in red (‘Hongxiangfei’) and black (‘Blackcurrant’ and ‘Sweet Sapphire’) raisins than in green raisins (‘Sultanina’). In ‘Sweet Sapphire’, D-saccharic acid, citrulline, and isovitexin were 26.02-, 37.86-, and 21.62-fold higher, respectively, than in ‘Sultanina’. In Figure 5B, 19 metabolites (including D-phenylalanine, levodopa, N-acetylphenylalanine, tryptamine, L-tryptophan, citric acid, 5-aminopentanoate, α,α-trehalose, D-myo-inositol 1,4-bisphosphate, L(−)-malic acid, dihydroxyacetone phosphate, dGMP, adenosine 5′-diphosphate, palmitic acid, LPC 17:0, LPA 21:1, LPC 16:0, gossypol, and caffeic acid) were significantly lower in red and black raisins than in green raisins. In Figure 5C, 11 metabolites (L-hydroxyproline, adenylosuccinic acid, D-raffinose, s7p, thymine, xanthosine, resveratrol, oenin chloride, luteolin, LPA 17:2, and 5-L-glutamyl-L-amino acid) were significantly more abundant in ‘Sweet Sapphire’ than in the other cultivars. Thymine, oenin chloride, and luteolin were 18.94-, 68.68-, and 25.69-fold higher, respectively, than in ‘Sultanina’. In Figure 5D, 13 metabolites exhibited varied distribution patterns. GABA and 2′-deoxyinosine 5′-monophosphate were higher in ‘Blackcurrant’ and ‘Sweet Sapphire’ than in ‘Hongxiangfei’ and ‘Sultanina’; the latter metabolite was 5.72- and 54.63-fold higher in ‘Blackcurrant’ and ‘Sweet Sapphire’, respectively, compared with ‘Sultanina’. Three metabolites (p-coumaric acid, uridine monophosphate, and rutin) were highest in ‘Blackcurrant’, while four others were highest in ‘Hongxiangfei’. Two metabolites were higher in both ‘Hongxiangfei’ and ‘Sweet Sapphire’ than in the remaining cultivars.

### 3.6. Correlation Analysis of the Relative Contents of Differential Metabolites in Raisins with Different Colors

The correlation analysis between the differential metabolites of raisins with different colors, identified based on positive and negative ion modes, and their antioxidant capacity is presented in Table 1. The total antioxidant capacity of raisins showed a significant positive correlation with 14 differential metabolites [(−)-epigallocatechin, syringetin, glycitin, coumestrol, trifolin, taxifolin, myricetin, dihydromyricetin, saccharopine, S-ribosyl-L-homocysteine, citrulline, urocanic acid, LPA 17:2, and 2′-deoxyinosine 5′-monophosphate], with correlation coefficients ranging from 0.951 to 1.000. Conversely, total antioxidant capacity was significantly negatively correlated with three differential metabolites (citric acid, cis-aconitic acid, and phosphocholine), with correlation coefficients from 0.965 to 1.000.

Polyphenol oxidase (PPO) activity was significantly positively correlated with 26 differential metabolites [(−)-epigallocatechin, syringetin, glycitin, coumestrol, petunidin-3-glucoside, trifolin, kuromanin, taxifolin, quercetin 3-O-sophoroside, laricitrin, L-dopa, epigallocatechin, myricetin, dihydromyricetin, oenin chloride, luteolin, L-saccharopine, S-ribosyl-L-homocysteine, citrulline, urocanic acid, L-hydroxyproline, UDP-galactose, LPA 17:2, L-pyroglutamic acid, thymine, and 2′-deoxyinosine 5′-monophosphate], with correlation coefficients ranging from 0.950 to 0.991.

The hydroxyl radical scavenging ability showed a significant negative correlation with 16 differential metabolites (L-tyrosine, 4-guanidinobutanoic acid, levodopa, L-tryptophan, inositol, L(−)-malic acid, dihydroxyacetone phosphate, LPC 18:0, PC 38:5, LPC 17:0, LPA 21:1, LPC 16:0, pheophorbide A, gossypol, cyclic AMP, and adenosine 5′-diphosphate), with correlation coefficients between 0.951 and 0.994. The superoxide anion scavenging ability was significantly negatively correlated with five metabolites (lycorine, 4-guanidinobutanoic acid, hexadecanedioic acid, gossypol, and cyclic AMP), with correlation coefficients ranging from 0.958 to 0.993.

### 3.7. Drivers of Antioxidant Capacity Variation in Black Raisins

**Figure 6:**
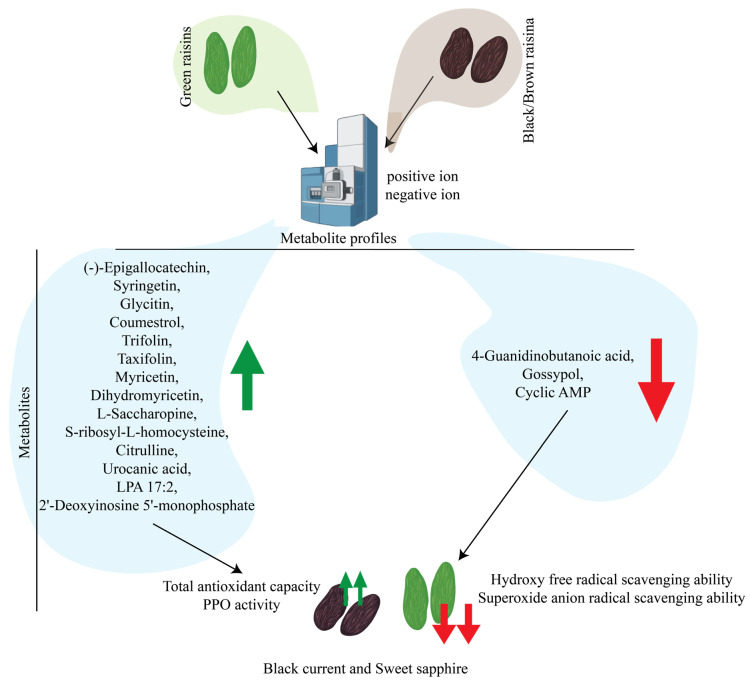
Mechanism analysis of the reasons for the differences in antioxidant capacity of black raisins. Green arrows shows high level and red arrow shows lower levels of metabolites.

Based on the above research, as shown in Figure 6, we infer that the relative contents of 14 different metabolites ([(−)-epigallocatechin, syringetin, glycitin, coumestrol, trifolin, taxifolin, myricetin, dihydromyricetin, L-saccharopine, S-ribosyl-L-homocysteine, citrulline, urocanic acid, LPA 17:2, and 2′-deoxyinosine 5′-monophosphate]) in black raisins (“Blackcurrant” and “Sweet Sapphire”) are relatively high, resulting in their high total antioxidant capacity and polyphenol oxidase activity. Furthermore, due to the relatively low content of the three differential metabolites (4-guanidinobutanoic acid, gossypol, and cyclic AMP) in black raisins, their hydroxyl radical scavenging ability and superoxide scavenging ability are relatively strong.

## 4. Discussion

Bioactive compounds extracted from natural sources benefit human health [30]. Plants are a rich source of antibacterial agents, with many modern medicines derived from plant-based resources [31]. Several studies have confirmed the efficacy of plant-derived compounds as antibacterial agents [32,33].

Raisins are notable for their high phenolic content and potent bioactivity. Among dried fruits, raisins exhibit the highest total phenolic content and antioxidant activity [34]. These antioxidant properties are primarily attributed to their phenolic compounds [35], making raisin extracts promising natural antioxidants for food systems. Additionally, a strong correlation exists between the phenolic content of raisins and their antibacterial activity [36]. Polyphenolic compounds in raisins are distributed approximately as follows: 5% in the juice, 1% in the pulp, 62% in the seeds, and the remainder in other components [37].

### 4.1. Black Raisins Have Higher Antioxidant Activity than Red or Green Raisins

Our study compared the antioxidant capacities of raisins of different colors, revealing significant differences between black (Blackcurrant and Sweet Sapphire), red (Hongxiangfei), and green (Sultanina) varieties. Black raisins exhibited significantly higher antioxidant capacities than red and green raisins, with ‘Sweet Sapphire’ demonstrating the highest total antioxidant capacity at 84.87 U/g, compared to 80.03 U/g for ‘Sultanina,’ the lowest. Additionally, Sweet Sapphire showed superior polyphenol oxidase (PPO) activity, hydroxyl radical scavenging ability, and superoxide anion scavenging ability, which were 1.13, 1.34, and 1.50 times higher, respectively, than those of ‘Sultanina.’

Antioxidants in raisins may reduce the risk of degenerative diseases associated with reactive oxygen species (ROS) [38]. Both in vitro and in vivo studies confirm raisins’ potent antioxidant and antibacterial activities, primarily driven by their phenolic content [39,40]. Correlation analysis of differential metabolites in raisins, identified using positive and negative ion models, showed that total antioxidant capacity was strongly positively correlated with eight secondary metabolites involved in biosynthesis pathways: (−)-epigallocatechin, syringetin, glycitin, coumestrol, trifolin, taxifolin, myricetin, and dihydromyricetin (Pearson’s correlation coefficients ranging from 0.951 for taxifolin to 1.000 for dihydromyricetin).

Phenolic compounds, synthesized via the shikimic acid/phenylpropanoid and acetate/malonate pathways, are key secondary metabolites with at least one phenolic unit. They are classified into subgroups, including phenolic acids, flavonoids, tannins, coumarins, lignans, quinones, stilbenes, and curcuminoids. Flavonoids, the largest subgroup, are widely present in the human diet and include anthocyanins (e.g., cyanidin, delphinidin, and malvidin), flavan-3-ols (e.g., catechin, epicatechin, and epigallocatechin), flavonols (e.g., quercetin and kaempferol), flavanones (e.g., hesperetin and naringenin), flavones (e.g., apigenin and luteolin), and chalcones [41]. Flavonoids act as effective antioxidants in vitro, neutralizing reactive molecules; repairing DNA damage; regulating nuclear receptors and gene expression; modulating enzyme activity; and influencing cell cycle, signaling pathways, and angiogenesis [42,43,44].

The higher antioxidant activity of black raisins, particularly ‘Sweet Sapphire,’ may be attributed to their elevated levels of (−)-epigallocatechin and myricetin, contributing to their superior total antioxidant capacity [20,45]. These findings highlight the potential of raisins, especially black varieties, as functional foods for reducing ROS-related disease risk. Flavonoids are a class of polyphenolic compounds widely present in fruits, vegetables, and medicinal plants and are renowned for their extensive health benefits and therapeutic potential. Among them, myricetin is a naturally occurring flavonol that has become a bioactive compound with significant pharmacological significance [46]. Myricetin exhibits various biological activities, including antioxidant, anti-inflammatory [47], anti-diabetes [48], antibacterial [49], anti-cancer [50], hepatoprotective [51], and neuroprotective effects. Therefore, consuming more black raisins is of great significance for the prevention and control of these diseases.

### 4.2. Metabolomic Dynamics Shift in Colored Raisins

In this study, as shown in Figure 4B and Figure 5B, 24 differential metabolites and 24 differential metabolites (2-hydroxycinnamic acid, L-tyrosine, L-arginine, lycorine, N-phenylacetylglycine, 4-guanidinobutanoic acid, 5-hydroxyindole-3-acetic acid, LPC 18:3, hexadecanedioic acid, LPC 14:0, LPC 18:0, LPC 38:5, ergocalciferol, adenosine, adenosine 5′-monophosphate, cyclic AMP, trigonelline, nicotinamide, desthiobiotin, 4-pyridoxate, D-panthenol, biliverdin, pheophorbide A, and inositol) were identified based on the positive-ion and negative-ion models, respectively. The relative contents of these differential metabolites were significantly higher in green raisins (‘Sultanina’) than in red raisins (‘Hongxiangfei’) and black raisins (‘Blackcurrant’ and ‘Sweet Sapphire’).

Notably, the relative content of inositol in green raisins (‘Sultanina’) was 32.00, 102.43, and 19.92 times higher than that in red raisins (‘Hongxiangfei’) and black raisins (‘Blackcurrant’ and ‘Sweet Sapphire’), respectively. Inositol is one of nine isomers of cyclohexanehexol, a small molecule with highly stable chemical properties and diverse functions. Myo-inositol (abbreviated as Ins for D-myo-inositol) is the most common form in biological systems, with at least five other forms (scyllo-, epi-, neo-, D-chiro-, and muco-inositols) also playing biological roles [52]. Myo-inositol, a stereoisomer of inositol, is essential in eukaryotes as a structural component of inositol phosphates, which serve as second messengers in intracellular signaling pathways. Inositol phosphates are critical for lipid signaling, cellular signal transduction, and membrane transport [53]. Inositol occurs naturally in fruits, vegetables, grains, and nuts [54].

Alterations in inositol metabolism are linked to various human diseases, primarily neuropsychiatric disorders, neurodegenerative diseases, and neurological conditions [55,56,57,58,59]. For instance, bipolar disorder—a debilitating neuropsychiatric condition that causes severe mood swings, reduces quality of life, and increases suicide risk [60]—shows a strong association with inositol levels. Clinical studies indicate that reducing inositol intake can alleviate the severity of bipolar disorder symptoms [61]. The relatively high inositol content in green raisins (‘Sultanina’) may offer potential benefits for treating human mental disorders, though further in-depth clinical research is required.

As shown in Figure 4D and Figure 5D, this study also identified 12 differential metabolites (trans-anethole, trans-cinnamaldehyde, pinocembrin, indole-3-lactic acid, indole-3-acetic acid, kynurenic acid, phenylacetylglutamine, phosphocholine, linoleic acid, thromboxane B2, 16(R)-HETE, and D-δ-tocopherol) and 4 differential metabolites (*cis*-aconitic acid, 11-dehydro thromboxane B2, lipoxin B4, and pantothenic acid) based on the positive-ion and negative-ion models, respectively. The contents of these metabolites were significantly higher in red raisins (‘Hongxiangfei’) than in black raisins (‘Blackcurrant and ’Sweet Sapphire’) and green raisins (‘Sultanina’). These compounds may have unique effects on human health and disease treatment, but additional research is needed to elucidate their functions in detail.

## 5. Conclusions

By using non-targeted metabolomics, we identified differential metabolites in different colored raisins. These results are of great significance for our understanding of the differences in antioxidant capacity among various colors of raisins. Antioxidant assays showed that black raisins had the highest antioxidant capacity, followed by red raisins, while green raisins had the lowest. Among black varieties, ‘Sweet Sapphire’ exhibited significantly higher antioxidant than ‘Blackcurrant’. Correlation analysis indicated that total antioxidant capacity was significantly positively correlated with 14 differential metabolites, with syringetin being particularly abundant in black raisins—likely contributing to their superior antioxidant activity. In addition, 43 and 16 differential metabolites were relatively abundant in green and red raisins, respectively, which may relate to the unique functional properties of these varieties.

## Figures and Tables

**Table 1:** Correlation analysis of differential metabolites of raisins of different colors identified based on positive and negative ion modes and their antioxidant capacity.

	TAC	PPOA	HFRSA	SARSA
TAC	1	0.949	0.638	0.754
PPOA	0.949	1	0.373	0.521
HFRSA	0.638	0.373	1	0.986 *
SARSA	0.754	0.521	0.986 *	1
(−)-Epigallocatechin	0.958 *	0.971 *	0.511	0.646
Syringetin	0.999 **	0.953 *	0.635	0.753
Glycitin	0.957 *	0.968 *	0.519	0.653
Coumestrol	0.968 *	0.965 *	0.551	0.681
Petunidin-3-glucoside	0.948	0.968 *	0.496	0.632
Trifolin	0.975 *	0.967 *	0.562	0.691
L-Tyrosine	−0.471	−0.171	−0.951 *	−0.901
L-Arginine	−0.376	−0.114	−0.92	−0.872
Lycorine	−0.907	−0.741	−0.899	−0.959 *
Kuromanin	0.95	0.972 *	0.487	0.624
Taxifolin	0.951 *	0.976 *	0.477	0.616
Quercetin 3-O-sophoroside	0.949	0.973 *	0.481	0.619
Laricitrin	0.947	0.973 *	0.475	0.614
L-Dopa	0.865	0.966 *	0.253	0.409
Epigallocatechin	0.807	0.950 *	0.093	0.257
Myricetin	0.994 **	0.967 *	0.595	0.72
Dihydromyricetin	1.000 **	0.954 *	0.632	0.75
Oenin chloride	0.949	0.972 *	0.485	0.623
Luteolin	0.949	0.971 *	0.487	0.625
L-Saccharopine	0.959 *	0.968 *	0.522	0.656
4-Guanidinobutanoic acid	−0.539	−0.27	−0.987 *	−0.958 *
S-ribosyl-L-homocysteine	0.965 *	0.976 *	0.512	0.647
Citrulline	0.955 *	0.968 *	0.512	0.647
Urocanic acid	0.980 *	0.972 *	0.558	0.688
Levodopa	−0.509	−0.233	−0.984 *	−0.948
L-Tryptophan	−0.459	−0.191	−0.961 *	−0.921
Citric acid	−0.965 *	−0.91	−0.685	−0.795
L-Hydroxyproline	0.844	0.957 *	0.216	0.373
Inositol	−0.433	−0.152	−0.965 *	−0.917
UDP-galactose	0.944	0.959 *	0.51	0.644
L(−)-Malic acid	−0.542	−0.251	−0.975 *	−0.938
Dihydroxyacetone phosphate	−0.438	−0.155	−0.967 *	−0.92
cis-Aconitic acid	−0.984 *	−0.947	−0.559	−0.679
Hexadecanedioic acid	−0.895	−0.713	−0.914	−0.966 *
LPC 18:0	−0.385	−0.08	−0.952 *	−0.888
PC 38:5	−0.465	−0.189	−.970 *	−0.929
Phosphocholine	−1.000 **	−0.954 *	−0.622	−0.74
LPC 17:0	−0.455	−0.178	−0.968 *	−0.925
LPA 21:1	−0.556	−0.296	−0.983 *	−0.960 *
LPC 16:0	−0.452	−0.173	−0.969 *	−0.924
LPA 17:2	0.955 *	0.991 **	0.437	0.581
Pheophorbide A	−0.501	−0.226	−0.980 *	−0.944
L-Pyroglutamic acid	0.846	0.960 *	0.212	0.37
Gossypol	−0.682	−0.436	−0.994 **	−0.993 **
Cyclic AMP	−0.575	−0.291	−0.989 *	−0.959 *
Adenosine 5′-Diphosphate	−0.393	−0.089	−0.956 *	−0.894
Thymine	0.95	0.976 *	0.473	0.612
2′-Deoxyinosine 5′-monophosphate	0.973 *	0.975 *	0.535	0.668

Notes: TAC: Total antioxidant capacity; PPOA: polyphenol oxidase activity; HFRSA: hydroxy free radical scavenging ability; SARSA: superoxide anion radical scavenging ability. * At the 0.05 level (double tail), the correlation is significant. ** At the 0.01 level (double tail), the correlation is significant.

## Data Availability

The original contributions presented in this study are included in the article/Supplementary Materials. Further inquiries can be directed to the corresponding author.

## Supplementary Materials

The following supporting information can be downloaded at https://www.mdpi.com/article/10.3390/antiox15030401/s1; Figure S1: Correlation diagrams of differential metabolites between ‘Hongxiangfei’ and ‘Sultanina’ raisins; Figure S2. Correlation diagrams of differential metabolites between ‘Blackcurrant’ and ‘Sultanina’ raisins; Figure S3. Correlation diagrams of differential metabolites between ‘Sweet Sapphire’ and ‘Sultanina’ raisins. Table S1. Screening results of differential metabolites. (Notes: A2: Sultanina; B2: Hongxiangfei; C2: Blackcurrant; D2: Sweet Sapphire).
