# Supplementary material for: Untargeted Metabolomics Reveals Color-Dependent Nutritional Variation in Raisins: Insights into Composition and Antioxidant Capacity
Source: Antioxidants (Basel). 2026 Mar 23;15(3):401. doi: 10.3390/antiox15030401 (PMC13023432; doi:10.3390/antiox15030401)
Supplement: Supplementary file 1 [file antioxidants-15-00401-s001.zip › Supplementary Figures-ZC.pdf]

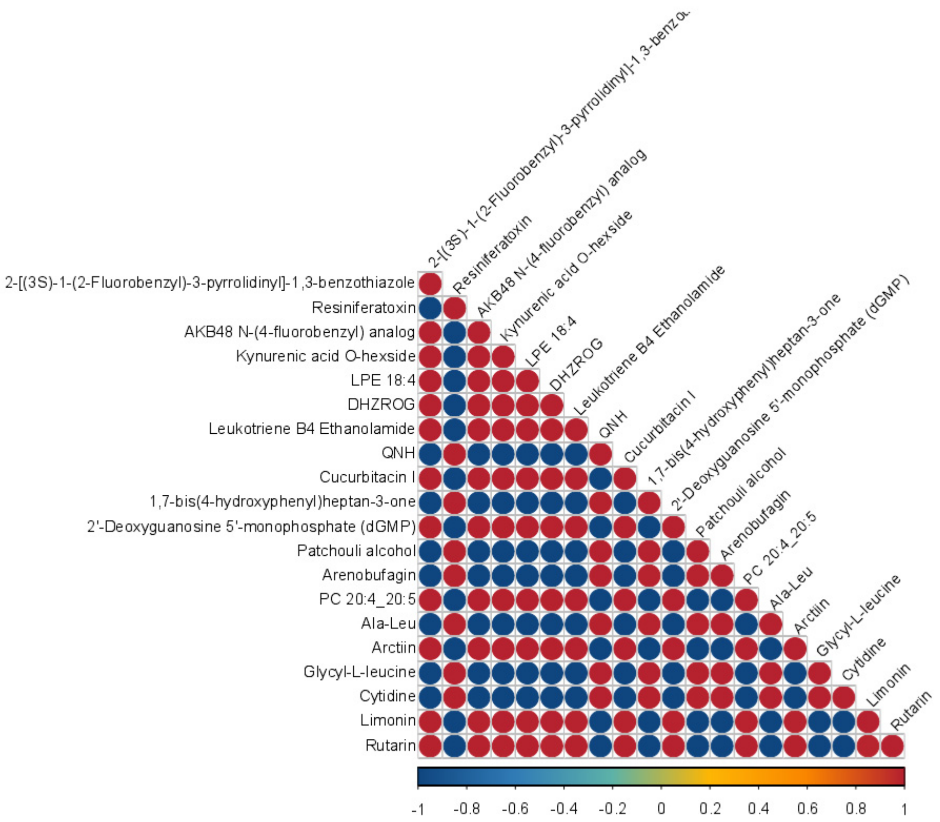

Figure S1. Correlation diagrams of differential metabolites between ‘Hongxiangfei’ and ‘Sultanina’ raisins

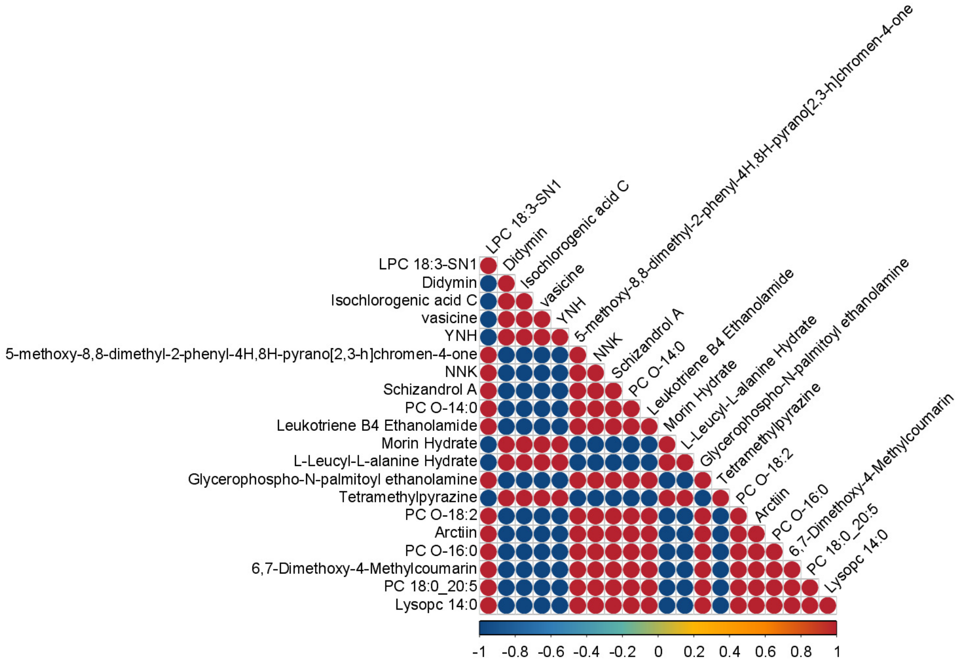

Figure S2. Correlation diagrams of differential metabolites between 'Blackcurrant' and 'Sultanina' raisins

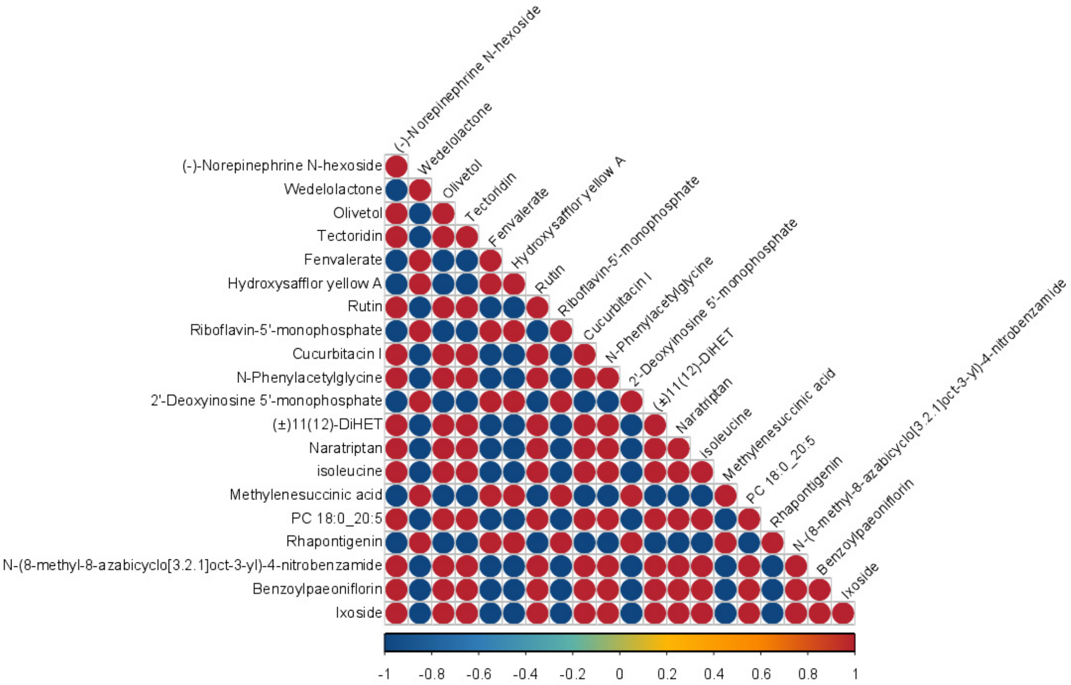

Figure S3. Correlation diagrams of differential metabolites between 'Sweet Sapphire' and 'Sultanina' raisins
